# Antibacterial and Proliferative Effects of NaOH-Coated Titanium, Zirconia, and Ceramic-Reinforced PEEK Dental Composites on Bone Marrow Mesenchymal Stem Cells

**DOI:** 10.3390/pharmaceutics15010098

**Published:** 2022-12-28

**Authors:** Artiom Lijnev, Jeevithan Elango, Vicente M. Gómez-López, Carlos Pérez-Albacete Martínez, José Manuel Granero Marín, José Eduardo Maté Sánchez De Val

**Affiliations:** 1 Department of Biomaterials Engineering, Faculty of Health Sciences, UCAM-Universidad Católica San Antonio de Murcia, Campus de los Jerónimos 135, Guadalupe, 30107 Murcia, Spain; 2 Center of Molecular Medicine and Diagnostics (COMManD), Department of Biochemistry, Saveetha Dental College and Hospitals, Saveetha Institute of Medical and Technical Sciences, Saveetha University, Chennai 600 077, India; 3 Green and Innovative Technologies for Food, Environment and Bioengineering Research Group (FEnBeT), Faculty of Pharmacy and Nutrition, UCAM-Universidad Católica San Antonio de Murcia, Campus de los Jerónimos 135, Guadalupe, 30107 Murcia, Spain; 4 Oral Surgery and Oral Implantology Department, UCAM-Universidad Católica San Antonio de Murcia, 30107 Murcia, Spain; 5 Department of Implant Dentistry, Faculty of Medicine and Dentistry, UCAM-Universidad Católica San Antonio de Murcia, Campus de los Jerónimos 135, Guadalupe, 30107 Murcia, Spain

**Keywords:** metallic implants, polyetheretherketone, surface modification, antibacterial activity, biocompatibility

## Abstract

Several metallic and polymer-based implants have been fabricated for orthopedic applications. For instance, titanium (Ti), zirconia (Zr), and polyetheretherketone (PEEK) are employed due to their excellent biocompatibility properties. Hence, the present study aimed to compare the functional and biological properties of these three biomaterials with surface modification. For this purpose, Ti, Zr, and ceramic-reinforced PEEK (CrPEEK) were coated with NaOH and tested for the biological response. Our results showed that the surface modification of these biomaterials significantly improved the water contact, protein adhesion, and bioactivity compared with uncoated samples. Among the NaOH-coated biomaterials, Ti and CrPEEK showed higher protein absorption than Zr. However, the mineral binding ability was higher in CrPEEK than in the other two biomaterials. Although the coating improved the functional properties, NaOH coating did not influence the antibacterial effect against *E. coli* and *S. aureus* in these biomaterials. Similar to the antibacterial effects, the NaOH coating did not contribute any significant changes in cell proliferation and cell loading, and CrPEEK showed better biocompatibility among the biomaterials. Therefore, this study concluded that the surface modification of biomaterials could potentially improve the functional properties but not the antibacterial and biocompatibility, and CrPEEK could be an alternative material to Ti and Zr with desirable qualities in orthopedic applications.

## 1. Introduction

Alloys-based metallic biomaterials are the most convincing implants that are widely used in orthopedic applications for the replacement of bone and dental tissues. Empirical evidence has already proved the biocompatibility, conductivity, and tissue regenerative properties of alloy-based materials. Among the biomedical alloys, titanium (Ti) and zirconia (Zr) are the best known materials in dental applications [1].

Ti, a well-known metal, is widely used in several practical applications as implants and abutments due to its excellent strength, light weight, and biocompatibility. More specifically, due to the excellent biocompatibility with the periodontal and bone tissues, Ti implants are considered as the most suitable materials in dental and bone regeneration. However, many scientific reports claimed the undesirable effects of Ti implants due to corrosion and poor esthetics, as well as the liberation of titanium ions [2,3,4,5,6]. In addition, the self-life of the Ti implant is potentially altered by environmental and mechanical factors such as wear, fatigue, and fretting corrosion [7]. Consequently, several alternative materials have been researched in recent times, and Zr could be a suitable alternative material to replace Ti [8,9]. Further, Zr implants exert a high level of biocompatibility [9], and the survival rates of zirconia implants are comparable with those of titanium ones [10].

Recently, several researchers have initiated the improvisation of the features of dental implants using other polymer materials. In this sense, polyetheretherketone (PEEK) has been widely studied as a suitable material [11,12,13,14,15]. Recently, Mishra and Chowdhary reported that PEEK-based implants could be an excellent system to enhance cell proliferation, adhesion, biocompatibility, and osteogenic properties and reduce peri-implant inflammations. They concluded that the PEEK could potentially replace the Ti in dental applications [3]. Interestingly, the excellent properties of PEEK in electrochemical corrosion resistance and reduction in weight make it a more beneficial material than metals. A semi-crystalline PEEK has exceptional properties such as a high melting point, high modulus, low density (1.32 g/cm^3^), stiffness, being insolvable in water, thermal stability, chemical stability, resistance to a wide range of chemicals (bases, hydrocarbons, organic solvents, and acids), processing performance, and good tribological properties. Among other biocompatible synthetic polymers, PEEK has the highest level of mechanical strength, chemical stability, and biocompatibility. Interestingly, the elastic modulus (3–4 MPa) of PEEK exactly resembles the human cancellous bone [14,16,17,18,19]. In addition, coating the metallic substrates with PEEK exhibited excellent adhesion properties, which proved the essential nature of PEEK in dental applications. Based on the above features, it has been substantially proved that PEEK can be used as a replacement for metals in dental applications with desirable properties.

However, Caballé-Serrano et al. reported a contradictory finding where they concluded that the PEEK had no better effect than Ti [20]. There are also reports stating similar evidence to support the conclusion of Caballé-Serrano et al. [21]. However, increasing clinical use of PEEK has revealed rather poor osseointegration, likely attributable to the inherent biological inertia of PEEK [14].

Although PEEK has several advantages, the bioinert property limits the use of PEEK in practical applications [22,23]. To support the above concept, Panayotov et al. [14] reported that the poor osseointegration of PEEK was attributable to the inherent biological inertia of PEEK. To overcome this issue, several authors have tried to modify the surface of PEEK by introducing antibacterial and bioactive agents through chemical treatment with the use of sulfuric acid [24] and NaOH [22,25]. Recently, the modified PEEK with 25% ceramic fillers called BioHPP (Bredent Group GmbH & Co.KG, Senden, Germany) has been reported to yield a better effect and overcome the drawbacks of PEEK in practical applications [26].

For instance, Guo et al. compared the effect of Ti, Zr, and PEEK with or without plasma and UV treatment in human dental pulp stem cells (hDPSCs) and human gingival fibroblasts and concluded that the UV and non-thermal plasma treatment improved the cellular attachment of Ti, Zr, and BioHPP PEEK and also upregulated the osteogenic differentiation of hDPSCs [26,27]. It was reported that the NaOH coating on PEEK, high-density polyethylene (HDPE), and ultra-high-molecular weight polyethylene (UHMWPE) films increased the nucleation and growth apatite layer [28]. To support the above novelty, another study also claimed that the bioactivity of vacuum-plasma-sprayed titanium on carbon fibre-reinforced PEEK was improved by NaOH coating [29]. However, none of the studies explored the coating effect of NaOH on these materials and the comparative effect of Ti, Zr, and ceramic-reinforced PEEK in mesenchymal stem cells. Hence, this study aimed to investigate the comparative effect of these samples with and without NaOH coating, which was the major novelty of this study. The present study hypothesized that the specific surface treatment of Ti, Zr, and CrPEEK with reducing agent NaOH would be expected to change the functional properties, thereby altering the biological response. In order to gain a better understanding of NaOH coating on the dental implant’s surface, the three specimens were coated with NaOH, and the surface adsorption ability, hydrophilic nature, antimicrobial properties, and in vitro biocompatibility were investigated using human bone mesenchymal stem cells (hMSCs).

## 2. Materials and Methods

### 2.1. Materials

The circular specimens with a diameter of 10 × 3 mm were prefabricated from Ti, Zr, and ceramic-reinforced polyetheretherketone (CrPEEK) (BIOHPP) by Bredent Group GmbH & Co.KG, Senden, Germany. BioHPP is a ceramic-reinforced high-performance polymer that displays great mechanical properties and biocompatibility [19]. All the reagents used in the experimental protocol were analytical reagents and obtained from local vendors unless otherwise specified.

### 2.2. Functionalization of Specimens

**Scheme 1:**
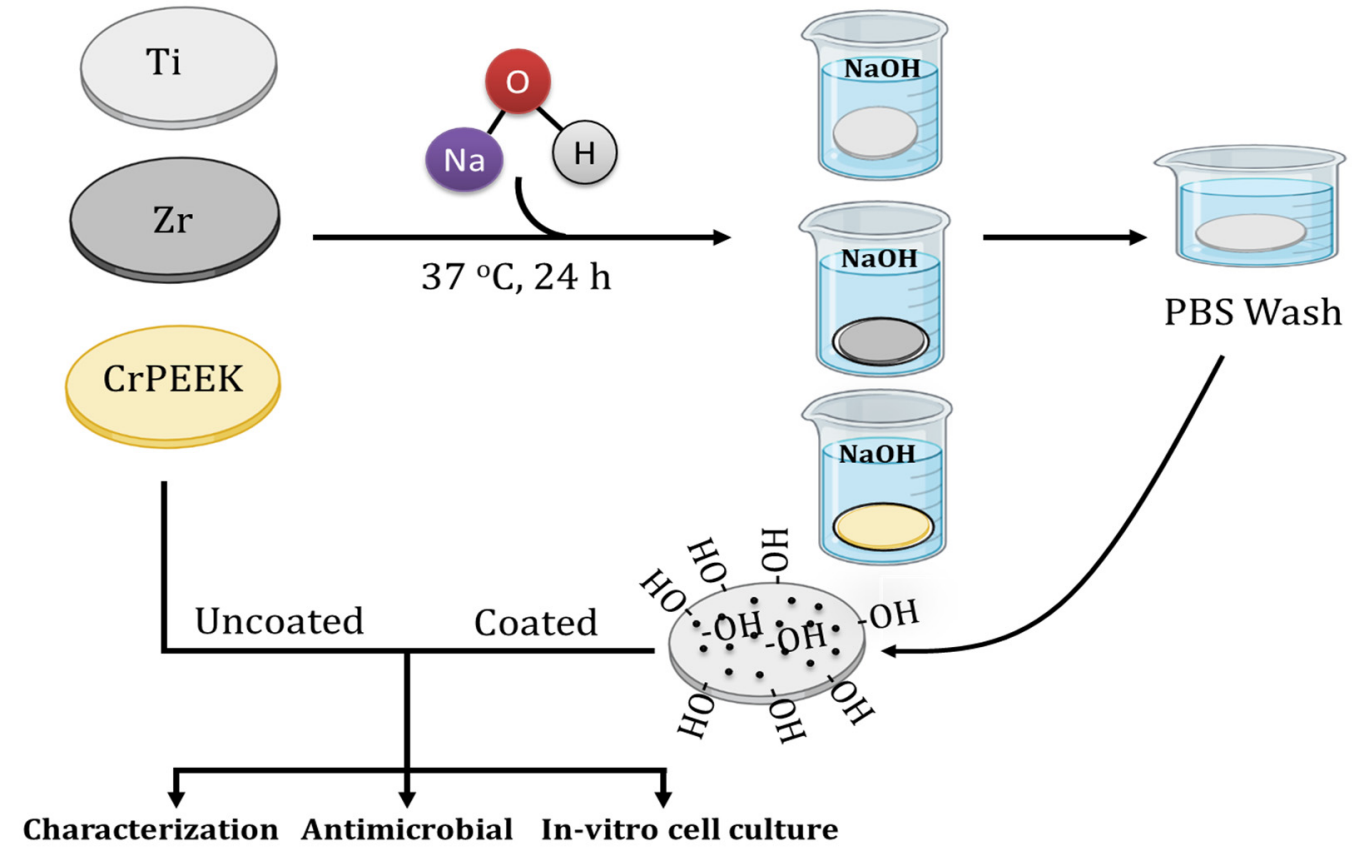
Schematic representation of surface coating of specimens with NaOH.

The surface of the specimen was functionalized by using sodium hydroxide (NaOH). To do this, the three specimens were incubated in 10 M NaOH for 24 h at 37 °C [30]. After incubation, the specimens were removed from the alkali solution and gently washed with phosphate-buffered saline (PBS) (Proquilab, Cartagena, Spain, Cat. No. 20012019) without disturbing the surface and dried. The uncoated specimens were considered as a control group. The coated and uncoated specimens were used for further experiments (Scheme 1).

### 2.3. Characterization of Specimens

#### 2.3.1. Protein Adsorption Ability

The protein adsorption ability of the specimen was determined by following our previous protocol [31]. Briefly, NaOH-coated and uncoated specimens were incubated in fetal bovine serum (FBS) (Lot No. 2445724RP, Gibco, Carlsbad, CA, USA) overnight at 37 °C. After incubation, the unbound and excessive proteins were gently washed out with PBS. In order to evaluate the adsorption of functional proteins on the surface, the specimens were stained on top with Coomassie brilliant blue G-250 (CBB) (Labclinics, Barcelona, Spain, Cat. No. 1752401) dye for 30 min. After 30 min, all the specimens were washed with PBS gently to remove the unbound dye and dried. The dye formed on the specimen was de-stained with a mixture of methanol/water/acetic acid, and the OD at 590 nm was measured using SpectraMax iD3 Multi-Mode Microplate Reader (Molecular Devices, LLC., San Jose, CA, USA).

#### 2.3.2. Bioactivity Assay

Evaluation of the apatite-forming ability of coated and uncoated specimens was conducted by using simulated body fluid (SBF) according to ISO/FDIS 23317 recommendations [32]. The SBF solution was prepared by following the standard method as described by Kokubo et al. [33]. Briefly, the samples were incubated in SBF at 37 °C for 28 days. After that, the samples were gently removed from SBF and washed with distilled water gently, and the amount of apatite deposition was measured using Alizarin Red stain by following our earlier protocol [31]. The quantitative analysis of stain on each sample was quantified using the UV-Vis spectrometric method at 450 nm using a SpectraMax iD3 Multi-Mode Microplate Reader.

#### 2.3.3. Contact Angle

The coated and uncoated samples were subjected to water and glycerol contact angle tests by using drop shape analysis at room temperature. Briefly, 5 µL of deionized water or 10 µL glycerol was dropped on the surface of the specimens, and the contact angle was measured at different time points, 0, 10, and 30 s. The samples were adapted to room temperature (with an RH of 60%) for moisture equilibrium prior to the experiment.

### 2.4. Scanning Electron Microscopy

The morphology of coated and uncoated specimens was visualized by using scanning electron microscopy in a SEM-Hitachi S-3500N device with energy-dispersive X-ray spectroscopy (EDS-INCA, Oxford Instruments Analytical, Wycombe, UK). The specimens were captured under different magnifications at 500 (100 μm), 1 (50 μm), 2 (20 μm), and 3 k (10 μm), respectively.

### 2.5. Antimicrobial Activity

The antimicrobial activities of coated and uncoated specimens were tested using the Gram-positive strain, *Staphylococcus aureus* (CECT 239), and Gram-negative strain, *Escherichia coli* (CECT 101), from the Spanish Type Culture Collection (CECT), Valencia, Spain. Freeze-dried bacteria were activated by culturing in Nutrient Broth from the activation kit of CECT and cultured for 24 h at 37 °C, followed by two 10 mL consecutive cultures in Nutrient Broth (Scharlau, Barcelona, Spain).

#### 2.5.1. Disc Diffusion Method

The strains from the second culture were spread using a sterile bent rod on a Standard Methods Agar (Panreac, Barcelona, Spain) culture plate (60 nm). In order to evaluate the effect of specimens on bacterial growth, after spreading, coated and uncoated specimens were placed into an agar plate and incubated for 24 h at 37 °C. The two antibiotics, penicillin and amphotericin-B (10 µL), were used to obtain the positive control. After incubation time, the zone of inhibition of coated and uncoated specimens was measured using an automatic Interscience scan 500 zone reader (Model:500, 436000S00871, Interscience International, Saint-Nom-la-Bretèche, Yvelines, Île-de-France, France).

#### 2.5.2. In Vitro Bacterial Attachment Assay

In order to investigate the bacterial attachment capacity on specimens, coated and uncoated specimens were placed into 24-well plates, and 400 µL from the second bacterial culture was added on top of the specimens and incubated for 24 h at 37 °C. After incubation time, the remaining unattached bacteria were removed gently with a PBS wash. Next, 3-(4,5-dimethylthiazol-2-yl)-2,5-diphenyltetrazolium bromide (MTT) (Cayman chemical company, Ref. 21795) reagent (400 µL) was added into wells for 2 h, and then the formazan dye was solubilized with 200 µL of dimethyl sulfoxide (DMSO) (Lot No. 52BC166BV, Sigma-Aldrich, St. Louis, MO, USA). The effect of bacteria attachment on specimens was spectrophotometrically quantified at 570 nm using a SpectraMax^®^ iD3 Multi-Mode Microplate Reader (Molecular Devices, LLC., San Jose, CA, USA).

### 2.6. In Vitro Cell Culture

Human bone Mesenchymal stem cells (hMSCs) (ATCC PCS-500-012, LGC Standards, Barcelona, Spain, Order Ref. No. 86605340) were used to evaluate the biocompatibility of the samples. The phenotyping and surface-specific marker expression of MSCs were reported in our earlier work [31]. The cells were cultured as per the standard cell culture protocol using complete Dulbecco’s Modified Eagle Medium (DMEM) (Proquilab, Cartagena, Spain, Ref. 11594416), consisting of 10% FBS and 1% antibiotic (penicillin and streptomycin P/S) in a 5% CO_2_ incubator at 37 °C. When the cells were at confluence around 70–80%, the medium was removed, cells were washed with PBS gently to remove the remaining medium or unbound cells, and trypsinization was performed using 0.25% trypsin-EDTA (Gibco, Carlsbad, CA, USA). The detached cells were centrifuged at 200× *g* for 5 min, and the cell pellet was redistributed with fresh medium for cell passage. Every time, the cell number was counted by using an automated Invitrogen cell counter (Countess 3 FL, Thermo Fisher Scientific, Waltham, MA, USA).

#### 2.6.1. Cell Loading Density

The coated and uncoated specimens were placed into 24-well plates with 800 µL of fresh medium in order to just cover the surface of the specimen. Then, the hMSC cells at a cell density of 5 × 10^4^ were carefully seeded on top of the specimens and incubated for 6 h. After the incubation period, the medium was removed, cells were trypsinized, and the total cell number was recorded using an automated Invitrogen cell counter (Invitrogen, Waltham, MA, USA). The cells seeded without specimens were considered as control.

#### 2.6.2. Cell Proliferation

The proliferation of hMSC cells in coated and uncoated specimens was evaluated. After seeding cells with a density of 5 × 10^4^ on top of the specimens as mentioned earlier, the culture plates were incubated for 7 days at 37 °C. The culture medium was changed every 2–3 days. After 7 days, the cells were trypsinized as mentioned earlier and counted by using an automated Invitrogen cell counter. The cells seeded without specimens were considered as the control.

#### 2.6.3. Fluorescence Microscope

The structure of the hMSC cells on top of the specimens was confirmed by fluorescence staining. Briefly, the cells were cultured on all specimens and trypsinized as mentioned before. Then, the cells (100 microliters) from each coated and uncoated specimen were collected and stained with fluorescence dye DAPI following standard protocol. In order to visualize the nucleolus of the cells, the images were captured by using fluorescence microscopy coupled with ZEISS Axiocam 305 mono (Axio Vert A1, Serial No. 3847016567, Carl Zeiss Microscopy GmbH, Suzhou, China).

### 2.7. Statistical Analysis

All the experiments were conducted in three independent setups, and the results were obtained in triplicate. The data were presented as mean standard deviation. The statistical significance was calculated by ANOVA using GraphPrism 9.0.1 (GraphPad Software Inc., San Diego, CA, USA). A *p*-value less than 0.05 (typically ≤0.05) was considered statistically significant.

## 3. Results

### 3.1. Adsorption Ability

**Figure 1:**
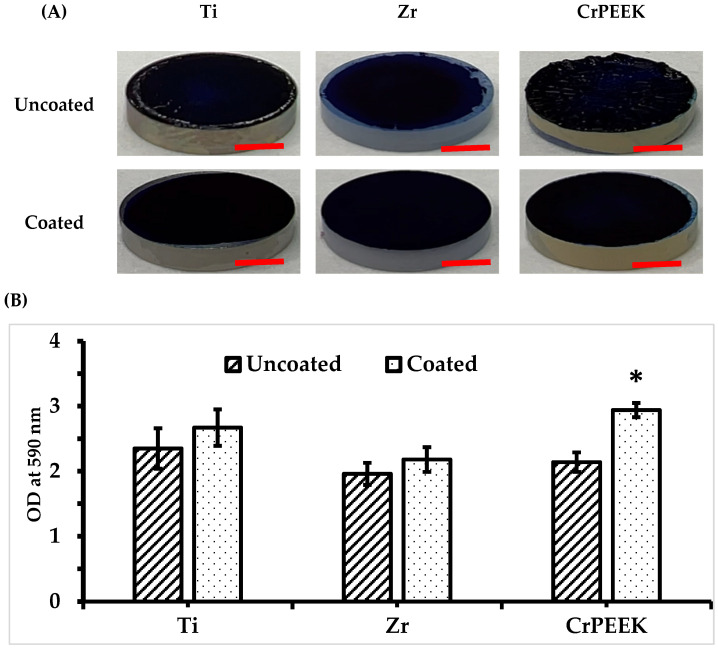
Coated and uncoated samples after CBB dye (**A**). A more uniform layer can be observed in coated samples and UV absorption of coated and uncoated samples analysed using a SpectraMax iD3 Multi-Mode Microplate Reader (**B**). Ti—Titanium; Zr—zirconium; CrPEEK—ceramic-reinforced PEEK. Scale bar: 0.3 cm. Values are mean + standard deviation, and * denotes statistical significance, *p* < 0.05 vs. uncoated.

The absorption ability of coated and uncoated samples was evaluated using the CBB dye method. In general, all coated and uncoated samples exhibited high adsorption ability to serum protein after FBS treatment. Notably, NaOH coating could potentially increase the ability of protein adsorption of the specimens (Figure 1A). The quantification result of protein by UV-Vis at 590 nm showed that the NaOH-coated active surface of the samples displayed an increased amount of protein adsorption compared with that of the uncoated control group (Figure 1B). Compared with the NaOH-coated specimens, the highest protein adsorption peak was observed in the CrPEEK sample, where coating showed a significant increase in protein adsorption ability. In contrast, among the uncoated samples, Ti displayed the highest value, followed by PEEK and Zr. The major changes in NaOH-coated groups were observed in CrPEEK samples, where the highest peak was observed, followed by Ti and Zr. Overall, the NaOH coating could significantly increase the protein binding ability of CrPEEK compared with other specimens, Ti and Zr.

### 3.2. Bioactivity Assay

**Figure 2:**
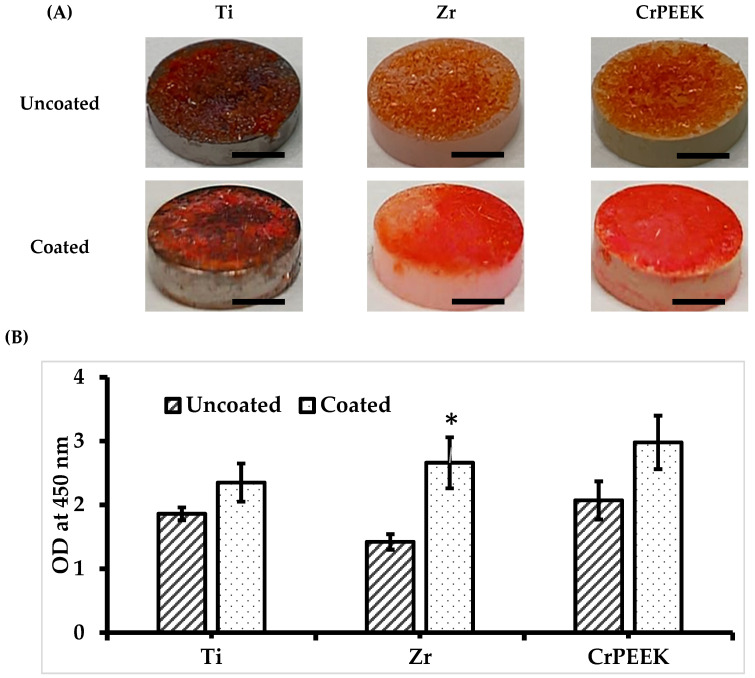
(**A**) Visualization of the apatite layer formed on each sample under Alizarin red stain. (**B**) Results obtained after quantitative analysis using the UV-Vis spectrometric method at 450 nm (SpectraMax iD3 Multi-Mode Microplate Reader). Ti—Titanium; Zr—zirconium; CrPEEK—ceramic-reinforced PEEK. Scale bar: 0.3 cm. Values are mean + standard deviation, and * denotes statistical significance, *p* < 0.05 vs. uncoated.

The mineral deposition (apatite layer formation) on the active surface of tested materials was measured using Alizarin red stain after SBF treatment for 30 days. In general, all the samples showed favourable mineralization on the active surface (Figure 2A). As we expected, similar to protein adsorption, the coated samples exhibited higher values in apatite layer formation compared with the uncoated group. The NaOH coating significantly improved the mineral deposition in the Zr materials compared with the other groups. However, among the specimens (both coated and uncoated), the CrPEEK samples displayed higher values in mineral deposition (Figure 2B).

### 3.3. Contact Angle

**Figure 3:**
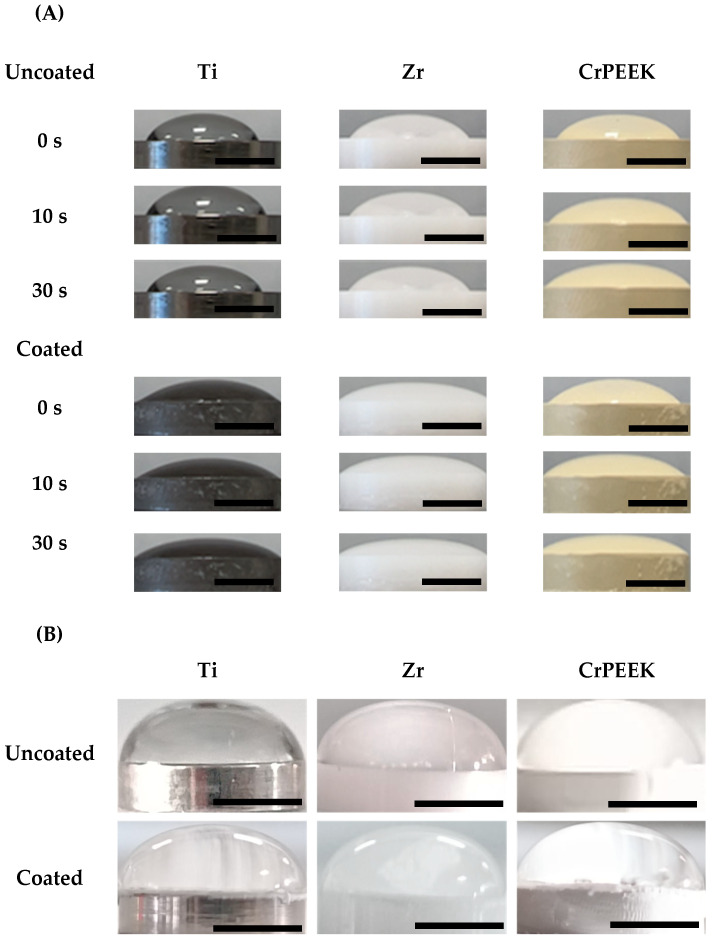
Contact angle behaviour of specimens. (**A**) Hydrophilic (water) measurement at different time points, 0, 10, and 30 s, and (**B**) hydrophobic (glycerol) at 30 s, respectively. Scale bar: 0.4 cm.

The hydrophilic and hydrophobic behaviour of the specimens were tested by using contact angle measurement [34]. NaOH treatment significantly increased the hydrophilicity of all active surface tested materials (Figure 3A), demonstrating great hydrophilic behaviour. However, CrPEEK showed similar results in both the coated and uncoated groups. Although the hydrophilic nature of the specimen was significantly altered by NaOH treatment, the hydrophobic behaviour of specimens was not affected by NaOH treatment compared with the uncoated specimens (Figure 3B).

### 3.4. Scanning Electron Microscopy

**Figure 4:**
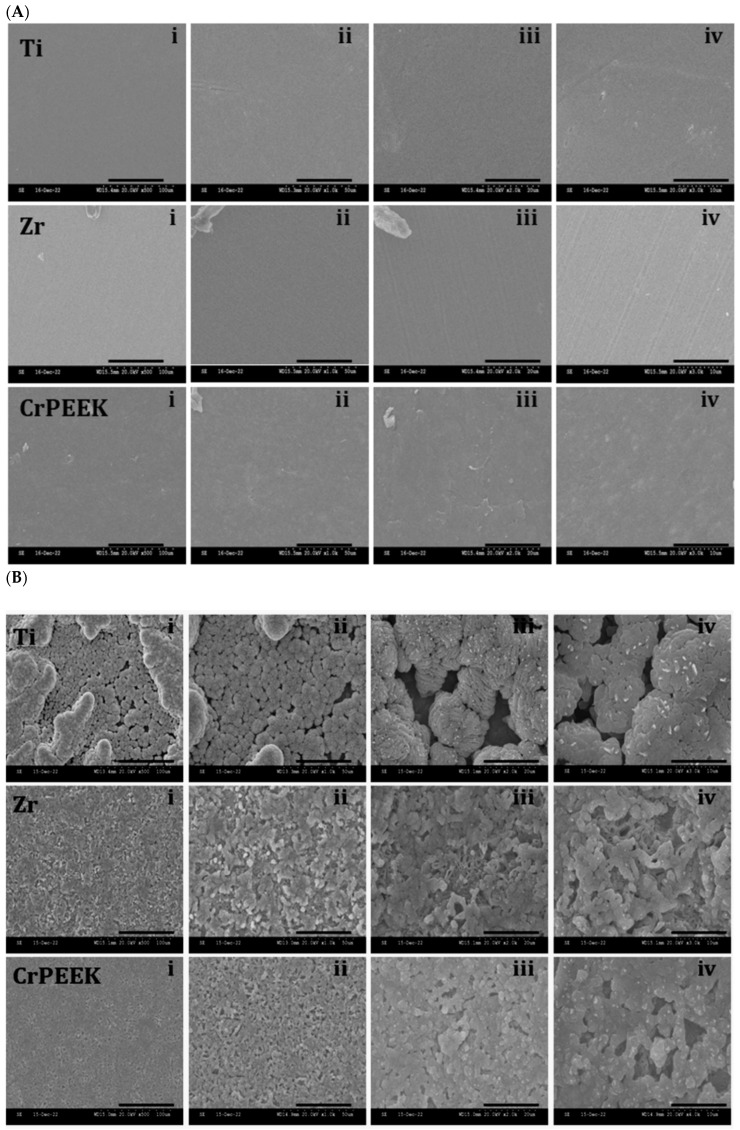
SEM microscopic surface characteristics of uncoated (**A**) and coated (**B**) specimens. The surface images of three specimens were captured at different magnifications: i—100; ii—50; iii—20; and iv—10 μm.

In order to investigate the pattern of surface coating, the uncoated and coated specimens were subjected to SEM. The results showed that all the uncoated specimens had a uniform homogenous surface; however, a smooth surface for Ti, a striated surface for Zr, and a rough surface for CrPEEK were seen at a higher magnification (10 μm) (Figure 4A). The surface morphology of the coated specimens confirmed the successful coating of NaOH on all three specimens’ surfaces (Figure 4B). Although the NaOH was successfully coated on specimens, the pattern of the NaOH coating was quite different between groups.

### 3.5. Disc Diffusion Method

**Figure 5:**
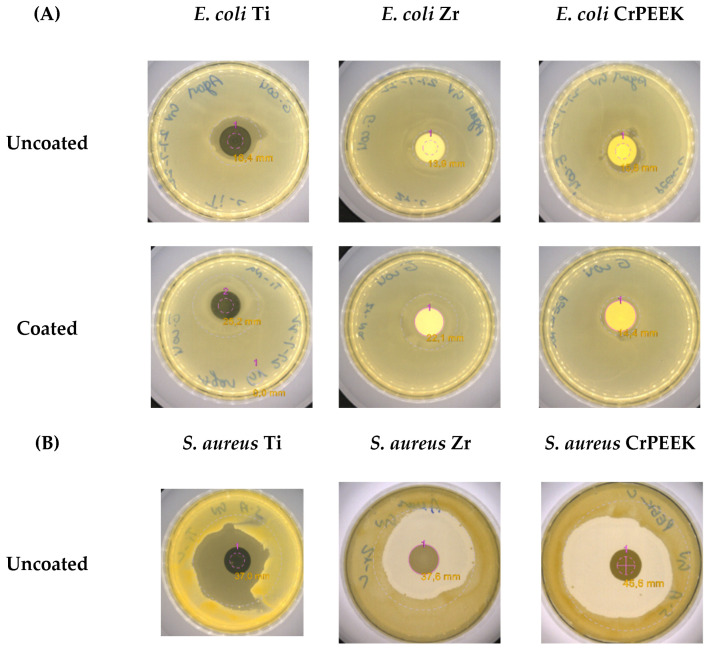

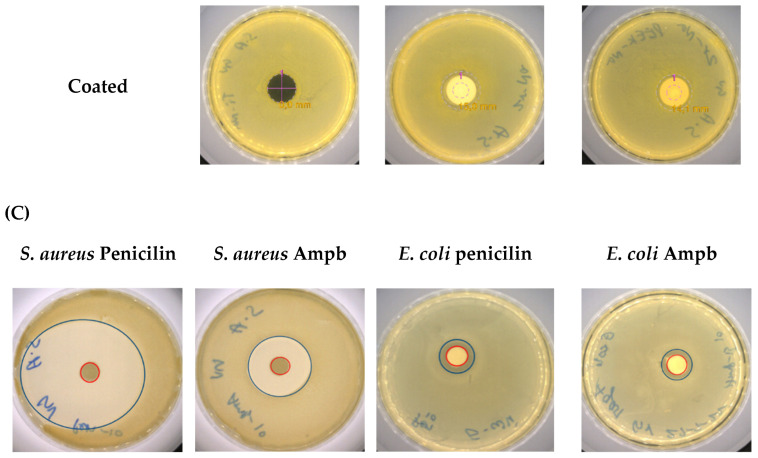
Macroscopic images of the antibacterial effect of the tested samples performing the disc diffusion method using *E. coli* and *S. aureus* strains. (**A**,**B**) Uncoated and coated samples and (**C**) positive control of *S. aureus* and *E. coli* using penicillin (10 μL) and amphotericin B (Ampb) (10 μL).

After the microbial incubation period, the susceptibility of tested materials in both groups was assessed by using two different bacterial strains, *S. aureus* and *E. coli*. Positive controls in terms of two antibiotics were also tested. Surprisingly, the results showed that the antimicrobial activity of coated materials decreased drastically compared with uncoated samples, and these changes in antimicrobial activity were specifically noticed in the samples tested with *S. aureus* strains (Figure 5).

The antimicrobial activity of uncoated samples against *E. coli* showed that the specimens made from Ti had a major inhibition effect compared with the other tested materials. There was no bacterial inhibition in Zr samples, and a low effect was shown in CrPEEK samples against *E. coli*. As previously mentioned, coating the active surface of the materials produced drastic changes in bacterial inhibition, especially since the specimen made from Ti lost its bacterial inhibition activity against *E. coli* strains (Figure 5).

On the contrary, different results were observed when uncoated samples were subjected to *S. aureus* strains. All samples showed bacterial inhibition activity, and CrPEEK samples exhibited the highest rate of inhibition followed by Zr and Ti, with no great difference between them (Table 1). As expected, amphotericin B showed a similar inhibition effect to penicillin against *E. coli*; however, the inhibition rate of penicillin was significantly higher than that of amphotericin B against *S. aureus* (Figure 5).

### 3.6. In Vitro Bacterial Attachment Assay

**Figure 6:**
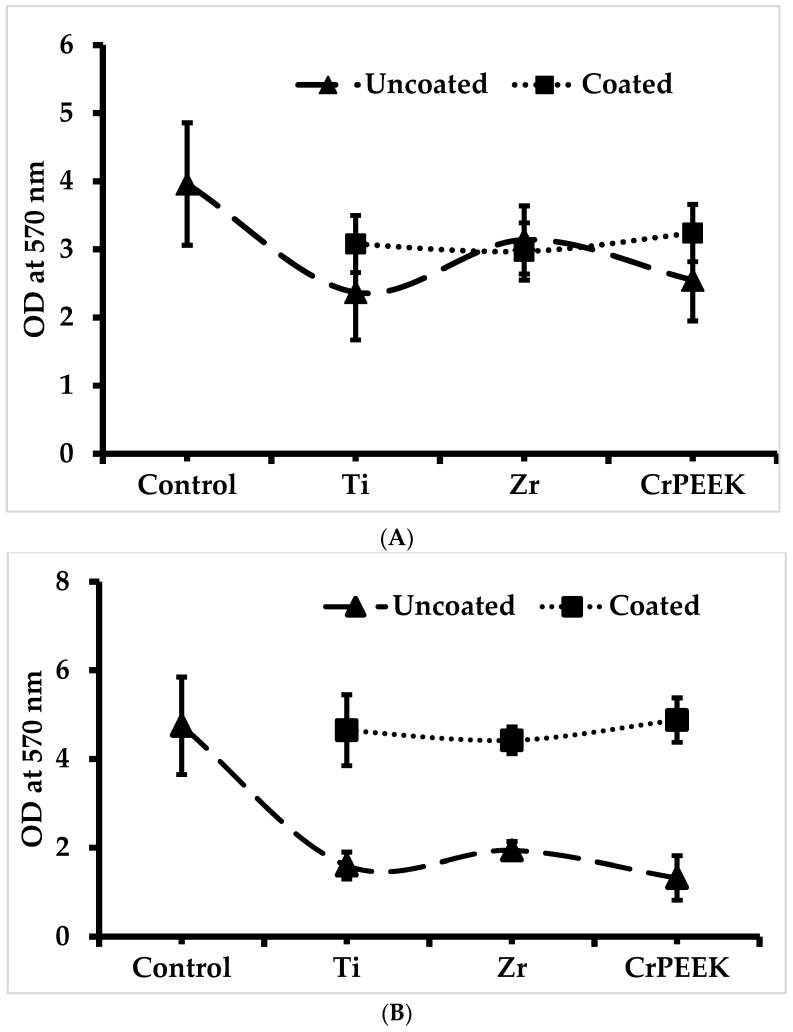
(**A**) Attachment assay of *S. aureus* on tested samples, with increased affinity observed after coating the samples with NaOH. Control—without samples. (**B**) Attachment assay of *E. coli* on tested samples. No significant increase in affinity level could be observed after coating the samples. Values are mean + standard deviation.

The ability of the specimens to attach bacteria was evaluated by culturing the respective strains over the discs in wells for 24 h and quantifying at 570 nm (Figure 6A,B). Treating the active surface of the samples with NaOH had an enhancing effect on bacteria attachment over the surface in comparison with uncoated samples, which substantially supported the antimicrobial activity of specimens by using the disc diffusion method. From two tested strains, *E. coli* showed greater attachment affinity to the sample surface in uncoated samples. In the case of NaOH coating, the samples showed no significant changes in attachment affinity with *E. coli*. However, a different scenario was observed in *S. aureus*, where treating the active surface of samples had a major effect on bacteria attachment and was increased more than twice compared with untreated specimens as shown in Figure 6A.

### 3.7. Cell Loading Density

**Figure 7:**
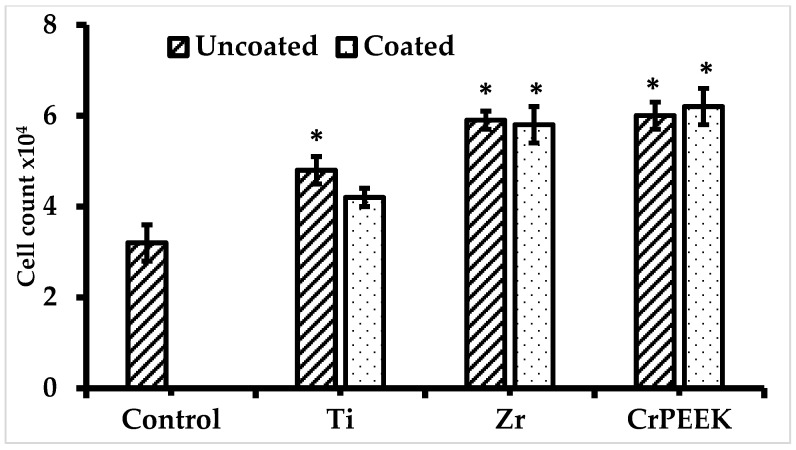
Cell loading ability of treated and untreated samples at 24 h incubation period. Values are mean + standard deviation, and * denotes statistical significance, *p* < 0.05 vs. control.

The total capacity of specimens in MSC seeding was assessed after a 24 h incubation period by following our previous protocol [31]. As shown in Figure 7, all the specimens showed great cell loading ability compared with the control. Among the specimens, CrPEEK showed the highest cell loading ability followed by Zr and Ti. The cells seeded alone in the control group had a loading density of 5 × 10^4^ cells after 24 h. Compared with the control, the NaOH-treated samples had decreased cell loading ability, except CrPEEK samples, where coating on an active surface improved cell loading ability. The NaOH coating increased the MSC loading rate in the CrPEEK specimen; however, it decreased the MSC loading rate in the Ti and Zr specimens.

### 3.8. Cell Proliferation

**Figure 8:**
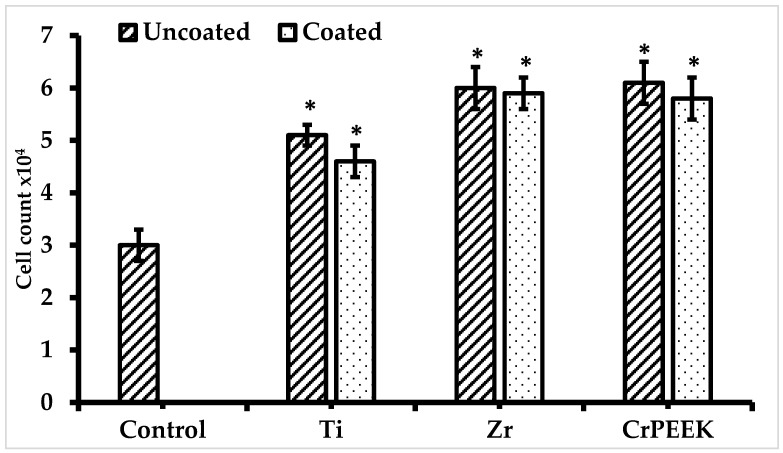
B-hMSC cell proliferation rate at one week. Control cells without biomaterials. TI, Zr, and CrPEEK represent cells cultured on titanium, zirconium, and ceramic-reinforced PEEK, respectively. Values are mean + standard deviation, and * denotes statistical significance, *p* < 0.05 vs. control.

The biocompatibility of specimens in MSCs proliferation was investigated by culturing cells over the disc surface for 7 days. As shown in Figure 8, the changes in the proliferation rate were not significantly affected by surface coating in the Ti, Zr, and CrPEEK samples. However, the proliferation rate of the coated surface of all tested materials was reduced on 7 days compared with uncoated groups, and the differences were statistically insignificant. Among the uncoated samples, CrPEEK and Zr had higher proliferation rates with a cell density of 6.3 × 10^4^ cells on 7 days. Compared with the control group (3.1 × 10^4^ cells), the coated and uncoated Zr and CrPEEK samples had a comparable proliferative effect.

### 3.9. Fluorescence Microscopy

The biocompatibility of coated and uncoated specimens in hMSC was further confirmed using a fluorescence microscope using a DAPI stain (Labclinics, Barcelona, Spain). The intensity of DAPI (nuclei) staining is a directional proposed for the efficiency of materials in cell proliferation and biocompatibility. The result showed that the fluorescent staining of the uncoated group loaded with MSCs was similar to a coated group (Supplementary Figure S1). Among the coated samples, the Zr group had higher fluorescence staining, which directly confirms the biocompatibility of Zr in hMSCs growth. On the other side, the uncoated CrPEEK group had higher intensity of DAPI stain compared with the other uncoated samples.

## 4. Discussion

In the present study, a wet chemical technique by enrichment with functional groups -OH (surface coating) was carried out in order to investigate the functional and antibacterial effects and biocompatibility of Ti, Zr, and CrPEEK specimens. All the specimens were coated with 10 M NaOH for 24 h at 37 °C based on the optimum conditions reported in earlier work [24]. In general, the coating process could affect the nature of specimens based on their properties. Therefore, it is important to choose the optimum experimental setup in order to avoid undesirable effects. In the present study, the absorption ability of protein in all three specimens was more pronounced by the surface coating of NaOH, especially in the PEEK sample. Similar to the present study, Escudeiro et al. reported that the surface coating of Zr implants with carbon increased the adsorption ability after treating with bovine serum albumin. Further, the authors concluded that the higher adsorption of protein by carbon coating proved the suitability of the implants in orthopedic applications [35]. Similarly, Jiang et al. reported that surface modification of Ti samples with NaOH promoted protein adsorption over time and speculated that the higher protein adsorption was due to the improved hydrophilicity of Ti samples after coating with NaOH. [36]. In addition, a report disclosed that the increased apatite formation in the PEEK samples was closely related to ion interaction between the surface of PEEK and SBF, in addition to hydrophilicity enhancement [37]. Likewise, the Zr samples coated with hydroxyapatite promoted bioactivity by increasing apatite layer formation [38]. Uchida et al. [39] stated that the higher apatite formation could be closely related to the formation of apatite nucleation induced by Zr-OH groups after SBF immersion of Zr-coated samples with NaOH. A few authors also suggested that apatite precipitation could be critically affected by the surface roughness and coating of the samples with NaOH [30,40]. It is reported that the hydroxyapatite-coated PEEK was more suitable for apatite formation [41]. Similar to the present result, Zheng et al. observed a firmly attached homogenize apatite layer on the functionalized PEEK surface after SBF treatment [42]. Our study confirmed that the higher deposition of apatite layers on the CrPEEK specimen demonstrated its suitability in the implantation process. Similar to the present results, Jiang et al. reported that the hydrophilic nature of the Ti specimen was increased by NaOH treatment [36]. From the wettability result, we opined that the surface roughness and superficial energy of coated samples were responsible for determining the hydrophilic nature of the specimen. The surface features of three specimens using SEM showed that the pattern of coating and crystal formation were potentially affected by the surface morphology of Ti, Zr, and CrPEEK. We speculate that the interaction of NaOH with these specimens and the arrangement of the coating might be the reason for the varying functional and biological responses of the specimens.

Among the specimens tested against two bacterial strains, the uncoated Zr had poor antibacterial activity. To support this finding, Aktuğ et al. reported that uncoated Zr exhibited greater adhesion to gram strains (*E. coli*) [38] and concluded that the microbial adhesion to the Zr surface was highly dependent on the hydrophobic–hydrophilic properties. We also found that the NaOH coating significantly increased the hydrophilicity of all tested samples and consequently displayed a decreased antimicrobial effect in all specimens coated with NaOH. The antibacterial result of the present study was in agreement with an earlier study reported by Gorth et al. who investigated the antibacterial effect of Ti, PEEK, and silicon nitride (Si_3_N_4_) against *Staphylococcus epidermidis*, *S. aureus*, *Pseudomonas aeruginosa*, *E. coli*, and *Enterococcus.* The authors explained the phenomenon that wettability (hydrophilic and hydrophobic), topography, protein adsorption, and other intrinsic biomaterial properties could potentially affect bacterial affinity, thereby influencing the antibacterial properties [43]. On the contrary, Lu et al. found that the nanostructured TiO_2_ on a carbon-fibre-reinforced polyetheretherketone surface could effectively reduce adhesion and growth of *S. aureus* and also had a partial influence on *E. coli* [44].

In general, the specimens did not show any toxic effect in hMSCs and, interestingly, showed a higher proliferative rate of hMSCs cultured on specimens than the control. Similarly, the in vitro biocompatibility of PEEK was previously tested in pre-osteoblast cells via attachment, spreading, and proliferation rate, and it was found that the PEEK specimen could significantly promote the formation of bone-like apatite. The authors also concluded that PEEK was a novel system to enhance bioactivity and thus expanded its possible applications in orthopedic and dental implants [42]. Another study compared the cell adhesion, viability, and proliferation of osteoblasts and gingival fibroblasts cultured on Zr, Ti, and PEEK specimens [45]. Recently, Blatt et al. cultured the human osteogenic cell line on Ti and PEEK with different surface modifications and suggested that the tested surfaces showed a positive influence on the differentiation and adherence of osteogenic cells [46]. From the in vitro cell culture study, the fabricated Zr, Ti, and CrPEEK specimens with or without surface modification showed excellent biocompatibility and also improved the cell proliferative rate of hMSCs. The SEM microstructural images clearly confirmed the successful coating of NaOH on the surfaces of all three specimens. We speculated that the deposition of hydroxyl ions on the surface altered the oxide layer of alloys by donating electrons and thereby enhanced the compatibility of specimens. To support this concept, a recent study investigated the efficiency of an AZ31 Mg alloy that was fabricated with plasma electrolytic oxidation in a 3.5 wt.% NaCl solution, in specific interactions with hexadecanoic acid and albumin molecules [47]. The above study also provided evidence that the binding efficiency with hexadecanoic acid and albumin was potentially altered due to the electrolytic oxidation of the AZ31 Mg alloy.

The major limitations of this study are that the biocompatibility and the osteogenic stimulatory effect of NaOH-coated specimens need to be verified by in vivo and in vitro models. The efficiency of the NaOH coating in these three samples needs to be compared with other types of coating such as plasma, ozone, and calcium in dental applications.

## 5. Conclusions

In the present study, the functional, antibacterial and biocompatible effects of three different biomaterials (Ti, Zr, and CrPEEK) with and without surface modification were investigated. Our results showed that the surface modification improved wettability, protein adhesion, and bioactivity; however, the beneficial effect of surface-modified biomaterials was not observed in the antibacterial activities of *E. coli* and *S. aureus*. In general, all three biomaterials showed biocompatibility, and the cells cultured on CrPEEK had a high proliferative rate compared with the other biomaterials; however, the effect was insignificant between biomaterials. Surprisingly, surface modification of the biomaterials did not contribute to promoting cell proliferation compared with uncoated samples. As expected, the cell loading ability of CrPEEK was more pronounced than Ti and Zr biomaterials. Overall, our study concluded that the CrPEEK could be an excellent alternative to Zr and Ti and a suitable material for the orthopedic applications.

## Figures and Tables

**Table 1:** Antibacterial effect of Ti, Zr, and CrPEEK samples against *E. coli* (nm) and *S. aureus* (nm) strains.

Samples	*E. coli* (nm)	*S. aureus* (nm)
Penicilin 10 µL	11.5 ± 1.4	25 ± 1.8
AmphotericinB 10µL	10.6 ± 0.7	21.7 ± 1.0
Ti	18.4 ± 0.7	37 ± 1.9 *
Zr	13.9 ± 1.1	37.6 ± 2.1 *
CrPEEK	15.8 ± 2.1	45.8 ± 3.5 *
Ti-NaOH	ND	ND
Zr-NaOH	ND	15.9 ± 1.1
CrPEEK-NaOH	14.4 ± 1.3	14.1 ± 0.8

ND—not detected. Ti—titanium; Zr—zirconia; CrPEEK—ceramic-reinforced PEEK; Ti-NaOH—coated Titanium; Zr-NaOH—coated zirconium; CrPEEK-NaOH—coated ceramic-reinforced PEEK. Values are mean + standard deviation, and * denotes statistical significance, *p* < 0.05.

## Data Availability

Not applicable.

## Supplementary Materials

The following supporting information can be downloaded at: https://www.mdpi.com/article/10.3390/pharmaceutics15010098/s1, Figure S1: Fluorescence staining (DAPI) of coated and uncoated specimens. Cells cultured on discs were trypsinized, and nuclei were visualized by DAPI stain.

## Abbreviations

Ti—titanium; Zr—zirconia; PEEK—polyetheretherketone; CrPEEK—ceramic-reinforced PEEK; *E. coli*—*Escherichia coli*; *S. aureus*—*Staphylococcus aureus*; hMSCs—human bone mesenchymal stem cells; PBS—phosphate-buffered saline; FBS—fetal bovine serum; CBB—Coomassie brilliant blue G-250; SBF—simulated body fluid; CECT—Spanish Type Culture Collection; MTT—3-(4,5-dimethylthiazol-2-yl)-2,5-diphenyltetrazolium bromide; DMSO—dimethyl sulfoxide; DMEM—Dulbecco’s Modified Eagle Medium; NaOH—sodium hydroxide.
