# Supplementary material for: Antibacterial and Proliferative Effects of NaOH-Coated Titanium, Zirconia, and Ceramic-Reinforced PEEK Dental Composites on Bone Marrow Mesenchymal Stem Cells
Source: Pharmaceutics. 2022 Dec 28;15(1):98. doi: 10.3390/pharmaceutics15010098 (PMC9863913; doi:10.3390/pharmaceutics15010098)
Supplement: Supplementary file 1 [file pharmaceutics-15-00098-s001.zip › pharmaceutics-2107870-supplementary.pdf]

Antibacterial and proliferative effects of NaOH-coated titanium, zirconia, and ceramic-reinforced PEEK dental composites on bone marrow mesenchymal stem cells

Artiom Lijnev [1], Jeevithan Elango [1,2,*], Vicente M. Gómez-López [3], Carlos Pérez-Albacete Martínez [4], José Manuel Granero Marín [5] and José Eduardo Maté Sánchez De Val [1,*]

1 Department of Biomaterials Engineering, Faculty of Health Sciences, UCAM-Universidad Católica San Antonio de Murcia, Campus de los Jerónimos 135, Guadalupe, 30107 Murcia, Spain
2 Center of Molecular Medicine and Diagnostics (COMManD), Department of Biochemistry, Saveetha Dental College and Hospitals, Saveetha Institute of Medical and Technical Sciences, Saveetha University, Chennai 600 077, India
3 Green and Innovative Technologies for Food, Environment and Bioengineering Research Group (FEnBeT), Faculty of Pharmacy and Nutrition, UCAM-Universidad Católica San Antonio de Murcia, Campus de los Jerónimos 135, Guadalupe, 30107 Murcia, Spain
4 Oral Surgery and Oral Implantology Department, UCAM-Universidad Católica San Antonio de Murcia, 30107 Murcia, Spain
5 Department of Implant Dentistry, Faculty of Medicine and Dentistry, UCAM-Universidad Católica San Antonio de Murcia, Campus de los Jerónimos 135, Guadalupe, 30107 Murcia, Spain
* Correspondence: srijeevithan@gmail.com or jelango@ucam.edu (J.E.); jemate@ucam.edu (J.E.M.S.D.V.); Tel.: +34-6-0359-7596 (J.E.)

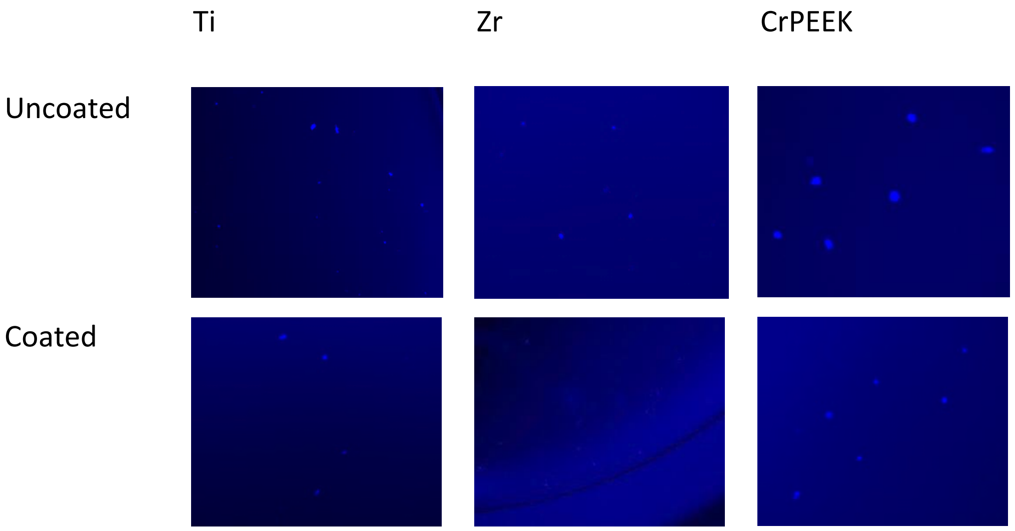

Supplementary Figure S1: Fluorescence staining (DAPI) of coated and uncoated specimens. Cells cultured on disks were trypsinized and nuclei were visualized by DAPI stain.
